# A Case of Wilms Tumor in a Primary Adrenal Mature Teratoma

**DOI:** 10.7759/cureus.41332

**Published:** 2023-07-03

**Authors:** Arwa Alahdal, Manal Bahkali, Noran M Abu-Ouf, Murad Alturkustani

**Affiliations:** 1 Pediatrics, King Abdulaziz University, Jeddah, SAU; 2 Pathology, King Abdulaziz University, Jeddah, SAU; 3 Pediatrics, King Abdulaziz University Faculty of Medicine, Jeddah, SAU

**Keywords:** somatic-type malignancy, primary adrenal teratoma, trisomy 21, wilms tumor, adrenal teratoma

## Abstract

We report the first known case of an adrenal teratoma containing a Wilms tumor component, in a 12-month-old girl with Trisomy 21. Despite adrenal teratomas being relatively uncommon, this particular instance raises interesting questions regarding the tumor origin, given the coexistence of both a teratoma and a Wilms tumor. Two main theories of development have been hypothesized, one of which suggests that the Wilms tumor may develop from a primary teratoma and the other proposing that the teratoma could originate from a primary Wilms tumor. Our case study leans toward the former, as the majority of the tumor displayed characteristics of a typical mature teratoma, with the Wilms component discovered as an incidental finding. Successful surgical intervention led to the gross total resection of the tumor. Twelve months post-resection, the patient remains free of recurrence. This report contributes to our understanding of these rare tumor types and underlines the importance of identifying the primary tumor to ensure appropriate management and treatment.

## Introduction

This report presents the first documented case of an adrenal teratoma with a Wilms tumor component. Primary adrenal teratomas are uncommon, with only 46 reported cases in adults [1] and seven cases in children [2]. Teratomas conjoined with Wilms tumors (nephroblastomas) have been detailed in 60 cases throughout the existing literature [3]. These tumors are most frequently located in the kidney and retroperitoneal regions, followed by the sacrococcygeal region, testis, ovaries, and mediastinum. Infrequent cases have been observed in the pineal gland, stomach, uterus, and vagina [3].

The coexistence of these two tumor types prompts speculations regarding their origin. The kidney, being the most common site for both these tumors, particularly for Wilms tumors and less so for teratomas, has led to postulations that the Wilms tumor might be the primary malignancy from which the teratoma develops [3]. However, the current case suggests an alternative hypothesis.

## Case presentation

A 12-month-old Yemeni girl with Trisomy 21 was admitted to the emergency department due to progressive abdominal distention, feeding intolerance, and constipation starting from the age of two months. There were no reports of fever, hematuria, bruising, petechiae, or bleeding from any orifices. She was born full term via vaginal delivery in a hospital and discharged in good condition. On physical examination, her height, weight, and head circumference were all below the 5th percentile, at 69 cm, 7 kg, and 43 cm, respectively. Clinical manifestations of Trisomy 21 were present. Her abdomen was distended with a large, non-tender, immobile, firm intra-abdominal mass primarily in the right quadrant, extending across the midline, measuring approximately 9x5 cm. Routine blood investigations including complete blood count (CBC), renal function, serum electrolyte, liver function, and thyroid function tests were within normal limits. However, the patient showed an elevated alpha-fetoprotein (AFP) level (11.8 ng/mL) with normal beta-human chorionic gonadotropin (BHCG). Urine analysis and cardiac echo were normal, and a chromosomal study confirmed Trisomy 21.

An abdominal computed tomography (CT) scan unveiled a sizable, heterogeneous retroperitoneal mass (9.8x10.7x12.7 cm) in the right suprarenal region; however, the adrenal gland was not visible. The mass demonstrated heterogeneous density and is displacing several neighboring structures, including the liver lobe, hepatic veins, gastroesophageal junction, liver hilum, portal vein, splenic vein, common bile duct, pancreatic head, and uncinate process. The inferior vena cava and bilateral renal veins are effaced, and the unremarkable right kidney is inferiorly and anterolaterally displaced. The mass is likely a teratoma.

The patient underwent an exploratory laparotomy under general anesthesia, revealing a large tumor in the right abdomen, displacing the diaphragm and abdominal contents. The tumor, closely adhered to the diaphragm and stretching the right renal vein, was fully mobilized and its blood supply ligated without significant bleeding or harm to nearby structures. Intentional rupture of the tumor's cystic part eased the tension on the renal vein. The tumor, along with a part of the suprarenal region, was successfully removed.

**Figure 1:**
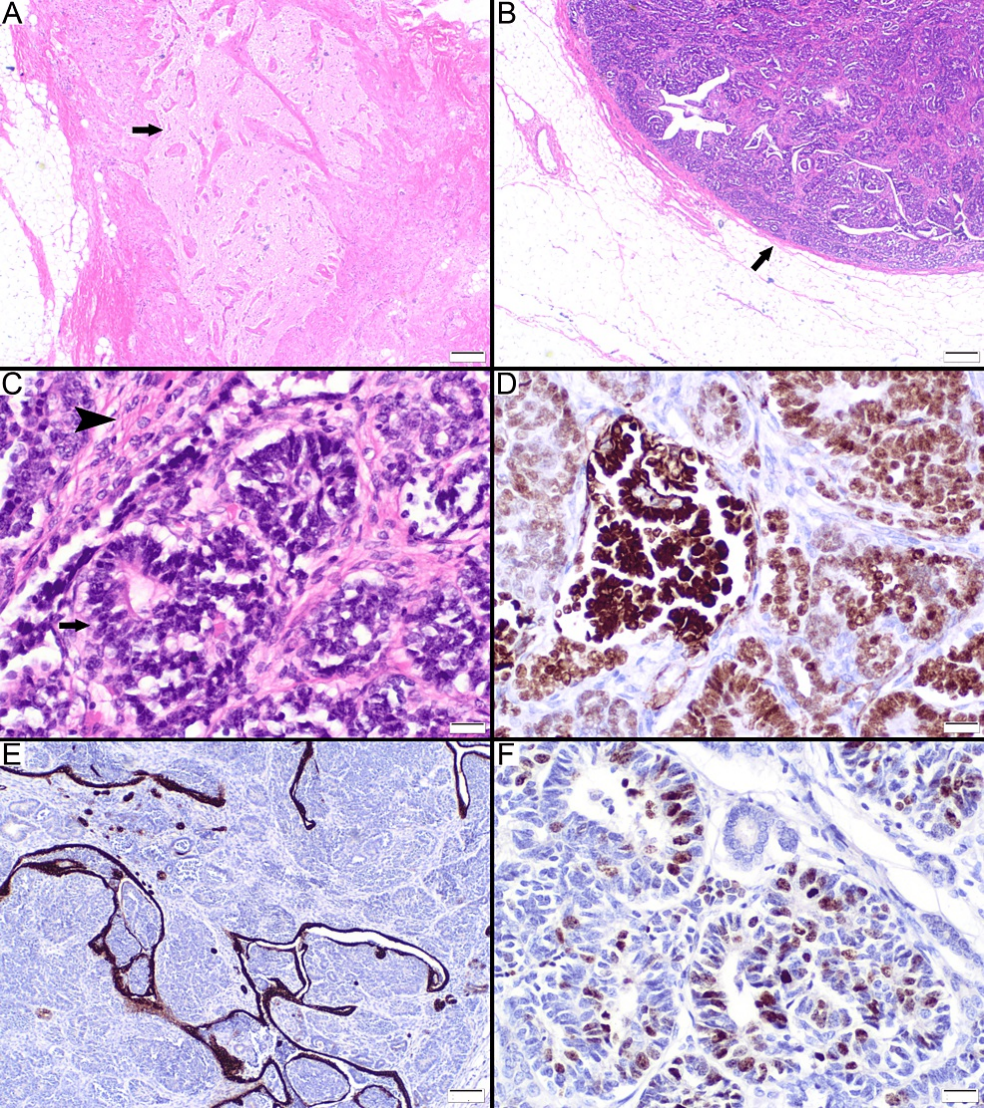
Histopathological features of the tumor (A) Histopathological examination revealing distinct mature components of the teratoma, including glial tissue (arrow) and fibrous and fatty tissue. (B) Small encapsulated and well-circumscribed immature renal tissue (arrow) observed within the tumor. (C) Triphasic Wilms tumor exhibiting immature glomeruli, nests of blastemal component, immature tubular epithelium (arrow), and mesenchymal component (arrowhead). (D) Immunostaining for WT1 highlighting the immature glomeruli and epithelium. (E) Immunostaining for CK7 demonstrating the presence of mature epithelial components. (F) Ki67 immunostaining indicating moderate nuclear positivity in the neoplastic immature epithelial tissue. Scale bar: 500 µm (A), 250 µm (B), 50 µm (C,D,F), 200 µm (E). Stains and magnification powers are detailed below: A is stained with hematoxylin and eosin (H&E) at a 20x magnification, B is stained with H&E at 40x, C is stained with H&E at 200x, D is stained with WT1 immunostain at 200x, E is stained with CK7 immunostain at 40x, and F is stained with Ki67 immunostain at 200x.

The pathology department received a 12x10x6.5 cm soft tissue mass, partially skin-covered. The mass was homogeneous, tan-yellow, and solid, with a focal cystic friable area measuring 1 cm in its largest diameter. Microscopic examination revealed various mature components, such as the skin, glial tissue, cartilage, fat, skeletal muscles, and different forms of epithelial glandular tissue (Figure 1, Panel A). There was no immature component. This array of mature tissues was indicative of a mature teratoma. An encapsulated, well-circumscribed immature renal tissue (Figure 1, Panel B) was observed, measuring 1.3 cm in its greatest dimension, nested within the teratoma, away from any surgical margin. The immature renal tissue consisted of immature glomeruli, nests of blastemal component, immature tubular epithelium, and mesenchymal component (Figure 1, Panel C), devoid of anaplastic features. The blastemal and epithelial component showed immunopositivity for Wilms' tumour gene 1 (WT1) immunostaining (Figure 1, Panel D), while only the mature epithelial component was immunopositive for cytokeratin 7 (CK7) (Figure 1, Panel E). Moderate positivity for Ki67 immunostaining was observed in the neoplastic cells of the immature epithelial tissue (Figure 1, Panel F). This segment of the tumor was diagnostic of a Wilms tumor emerging from the mature teratoma.

The patient received no additional treatment. At the 12-month follow-up post-resection, she was thriving well with normal vital signs, and no palpable abdominal mass. Laboratory tests, including CBC, BHCG, and AFP, were normal, as was the abdominal ultrasound.

## Discussion

Teratomas, falling under the non-germinomatous germ cell tumor classification, are identified by both their histological makeup (mature or immature) and their anatomical placement (gonadal or extragonadal) [4]. A unique subset of teratomas is associated with somatic-type malignancy, which refers to the presence of a distinct malignancy within a teratoma [5]. This condition is also referred to as the malignant transformation of a germ cell tumor. However, as Hwang et al. highlighted, this terminology is misleading, given that a teratoma in itself is a malignant disease with the ability to metastasize and lead to death [5]. Wilms tumors that develop within a teratoma can meet the criteria to be classified into this category. In fact, they were the somatic malignant component in 3 out of 63 testicular germ cell tumors, according to the same source [5].

Sacrococcygeal teratoma is the most commonly observed teratoma in children, with ovarian teratomas being the second most prevalent in this age group [4]. Conversely, adrenal teratomas are relatively infrequent, with varying incidences reported in different studies. One research institution reported a mere three cases among 7706 patients undergoing adrenalectomy over six decades [6]. Another study found two instances among 338 adrenalectomy patients during their research period [2]. A review of the existing literature yielded a range of cases, peaking at 46 adult cases [1]. Most of these involved mature teratomas, with no instances of Wilms tumor reported.

There have been approximately 60 reported instances of Wilms tumors concomitant with teratomas, but none have been located in the adrenal gland [3]. The distribution of these 60 cases spans various locations: kidney (34 cases), retroperitoneal (six cases), sacrococcygeal region (four cases), testis (four cases), ovary (two cases), mediastinum (two cases), and abdomen (two cases). In addition, single instances have been reported in the pineal region, thorax, stomach, ureteropelvic junction, uterus, and vagina [3].

Two potential developmental paths for these tumors have been hypothesized: either a nephroblastoma emerges from a primary teratoma or a teratoma originates from a primary Wilms tumor. The latter hypothesis is reinforced by the fact that more than half of these cases involve the kidneys, the primary site for Wilms tumors [3]. Extrarenal instances can potentially be explained by the possibility of Wilms tumors originating from the ectopic metanephric blastemal or primitive mesodermal tissue, known as Connheim's cell rest [7]. The potential existence of a concomitant tumor, particularly in this case, given the patient's Trisomy 21, was considered. However, this possibility was deemed minimal since both components of the tumor were present within the same mass. While we cannot definitively exclude the chance that the Wilms component might represent metastasis from another tumor, the imaging examination and clinical follow-up did not reveal any additional primary tumors, making this likelihood improbable.

In this specific case, it is highly likely that the tumor is primarily a teratoma containing a small embedded component of Wilms tumor. This deduction is based on the fact that the majority of the tumor corresponds to a typical mature teratoma in both macroscopic and microscopic examinations, with the Wilms tumor component being an unexpected incidental discovery. Identifying the primary tumor is crucial, as the management strategies differ for Wilms tumors and mature teratomas hosting a somatic malignancy.

## Conclusions

Our report represents the first documented occurrence of an adrenal teratoma containing a Wilms tumor component. It suggests that the teratoma might be the primary tumor in this rare form of coexistence, deviating from prior hypotheses. The identification of the primary tumor in such complex scenarios is critical due to differing management strategies for mature teratomas with somatic malignancies, such as Wilms tumor, and Wilms tumor itself. Importantly, successful surgical intervention resulted in the gross total resection of the tumor and no recurrence 12 months post-surgery. This case adds to the limited body of literature on adrenal teratomas and highlights the potential for rare, unexpected findings within these tumors. Moreover, it underscores the need for careful histopathological examinations in these rare cases. The study invites further research and case collection to better understand the origin and development of such tumor.
